# Group clinics for young adults with diabetes in an ethnically diverse, socioeconomically deprived setting (TOGETHER study): protocol for a realist review, co-design and mixed methods, participatory evaluation of a new care model

**DOI:** 10.1136/bmjopen-2017-017363

**Published:** 2017-06-21

**Authors:** Chrysanthi Papoutsi, Dougal Hargreaves, Grainne Colligan, Ann Hagell, Anita Patel, Desirée Campbell-Richards, Russell M Viner, Shanti Vijayaraghavan, Martin Marshall, Trisha Greenhalgh, Sarah Finer

**Affiliations:** 1 Nuffield Department of Primary Care Health Sciences, University of Oxford, Oxford, UK; 2 UCL Great Ormond St. Institute of Child Health, University College London, London, UK; 3 Centre for Primary Care and Public Health, Blizard Institute, Barts and The London School of Medicine and Dentistry, Queen Mary University of London, London, UK; 4 Association for Young People’s Health, London, UK; 5 Barts Health NHS Trust, London, UK; 6 Department of Primary Care and Population Health, University College London, London, UK

**Keywords:** group clinics, diabetes, young adults, realist review, evaluation, co-design.

## Abstract

**Introduction:**

Young adults with diabetes often report dissatisfaction with care and have poor diabetes-related health outcomes. As diabetes prevalence continues to rise, group-based care could provide a sustainable alternative to traditional one-to-one consultations, by engaging young people through life stage-, context- and culturally-sensitive approaches. In this study, we will co-design and evaluate a group-based care model for young adults with diabetes and complex health and social needs in socioeconomically deprived areas.

**Methods and analysis:**

This participatory study will include three phases. In phase 1, we will carry out a realist review to synthesise the literature on group-based care for young adults with diabetes. This theory-driven understanding will provide the basis for phase 2, where we will draw on experience-based co-design methodologies to develop a new, group-based care model for young adults (aged <25 years, under the care of adult diabetes services). In phase 3, we will use a researcher-in-residence approach to implement and evaluate the co-designed group clinic model and compare with traditional care. We will employ qualitative (observations in clinics, patient and staff interviews and document analysis) and quantitative methods (eg, biological markers, patient enablement instrument and diabetes distress scale), including a cost analysis.

**Ethics and dissemination:**

National Health Service ethics approval has been granted (reference 17/NI/0019). The project will directly inform service redesign to better meet the needs of young adults with diabetes in socioeconomically deprived areas and may guide a possible cluster-randomised trial, powered to clinical and cost-effectiveness outcomes. Findings from this study may be transferable to other long-term conditions and/or age groups. Project outputs will include briefing statements, summaries and academic papers, tailored for different audiences, including people living with diabetes, clinicians, policy makers and strategic decision makers.

**Registration details:**

PROSPERO (CRD42017058726).

**Strengths and limitations of this study**

- This study will draw on the strengths of group-based care to address the complex needs of young adults (aged 16–25 years) living with diabetes in ethnically diverse, socioeconomically deprived backgrounds.
- Cultural, developmental and practical considerations will be taken in account as part of iterative co-design and participatory evaluation of the new care model.
- A theory-driven, realist review of the literature will further inform the co-design process and will strengthen the transferability of findings.
- The project does not intend to generate an effect size, but findings may inform the design of a future cluster-randomised controlled trial.

## Introduction

Diabetes has been described as one of the most significant global public health challenges of our time.^1^ Over the last four decades, the global prevalence of both type 1 and type 2 diabetes has increased sharply to reach an estimated 8.5% in the adult population in 2014.^2^ In the UK, recent figures place the number of adults living with diabetes at 4.5 million, of whom around 1 million remained undiagnosed in 2016.^3^ This has raised the cost of diabetes care to 10% of the annual National Health Service (NHS) budget and has highlighted an urgent need to investigate different ways of delivering diabetes prevention and care.^4 5^

Despite growing emphasis on improving diabetes prevention and care in the adult population, less attention has been paid to young people living with diabetes. In England and Wales, over 27 000 children and young people with diabetes receive care in paediatric diabetes units,^6^ and increasing numbers (poorly recorded) of adolescents and young adults are living with the condition. Young people constitute an important group for early intervention as good self-management practices internalised at a young age could persist throughout adulthood and reduce the risk of lifetime complications, prevent early mortality and lower costs for the health service.^7 8^ Young people of today will grow up to constitute the estimated 642 million living with diabetes worldwide in 2040.^1^ Yet, in 2014/2015, only one quarter of children and young people with diabetes in England and Wales achieved recommended blood glucose control (HbA1c <58 mmol/mol as per the National Institute for Health and Care Excellence 2004 target) and a similarly low proportion received all recommended care processes, with high variability between care providers.^6^ This is reinforced by research showing that mortality among young adults with diabetes in the UK is worse than in other European countries and rose significantly between 1990 and 2010.^9^ Diabetes is also known to have serious consequences in those diagnosed in childhood: diabetes-related complications (such as kidney and eye diseases) were seen in 1 in 3 of those with type 1 diabetes, and 3 in 4 with type 1 diabetes in their early 20s, within 8 years of diagnosis.^10^

Barriers to accessing healthcare for younger people have been well described, including lack of developmentally appropriate consultations, fear of being judged and stigmatised, lack of equitable access to services and diabetes-related distress.^11 12^ Published data show that young adults report the worst NHS experience of any age group and have distinct healthcare needs and priorities compared with other age groups.^13 14^ This may be even more important for young people from socioeconomically deprived areas who achieve worse blood glucose control and present with more complications compared with more affluent areas and for those in ethnic minority groups who are disproportionately affected by type 2 diabetes.^6 15^

The TOGETHER project will employ participatory methods to co-design, deliver and evaluate a model for diabetes care that addresses the needs of young people (aged 16–25 years) from socioeconomically deprived backgrounds. This model will draw on the strengths of group-based care, involving consultations and education sessions delivered by a multidisciplinary team and attended concurrently by a group of patients, in contrast to traditional one-to-one, consultant-led care.

Previous research has shown that group-based care in adults with diabetes resulted in better glycaemic control, problem-solving ability and quality of life and reduced time commitment for clinicians, compared with standard one-to-one consultations.^16 17^ Systematic reviews on group care for diabetes showed additional benefits for clinical and patient-reported outcomes.^18 19^ Further trials applying group-based care to diabetes and other conditions are underway with some work focusing on children and adolescents.^20–22^ However, these studies are limited to health but not social care needs, they have not utilised co-design processes or extensive user engagement and they are primarily targeting patient groups other than adolescents and young adults (aged 16–25 years) in ethnically diverse, socioeconomically deprived backgrounds.

Our research seeks to address these uncertainties, including whether group-based care could be successfully adapted for this younger patient group and whether rigorous co-design of a new model of care can enhance acceptability and engagement. Furthermore, we will explore whether the positive impact of group-based education such as DAFNE (Dose-Adjustment for Normal Eating - a structured education programme for people withtype 1 diabetes) and DESMOND (Diabetes Education and Self Management for Ongoing and Newly Diagnosed - a structured education programme for people with type 2 diabetes)^23 24^ as well as supporting evidence from studies of peer support^25 26^ could be harnessed in group clinics for young adults to improve engagement with diabetes care and self-management in the wider context of individual, family, peer and social influences.^27–29^ This work will extend previous learning on the role of peer support groups and mentoring as a way to address complex health and social care issues.^29–31^

The TOGETHER project will be driven by the following aims and research questions:

### Aims

1. To explore the scope, feasibility, impact and potential scalability of group clinics for young adults with diabetes and complex health and social care needs.
2. To contribute to NHS service re-design and improve care for people from hard-to-reach groups with long-term conditions.

### Research questions

1. How and to what extent might an innovative, co-designed group clinic-based care model meet the complex health and social needs of young people with diabetes?
2. Could a group approach help support diabetes self-management? If so, what do the experiences of participants, the functioning of the group and the wider context in which the new model takes place tell us about its mechanisms of action?
3. What are the feasibility, acceptability, costs and impact on outcomes of introducing group clinics for their users and stakeholders? What is the organisational impact of this model to the NHS and other stakeholders?
4. What would be the optimal size and study design of a cluster-randomised controlled study to evaluate the clinical benefit and cost-effectiveness of offering group clinics to all suitable young adults with diabetes? What other factors should be considered when planning such an RCT (eg, factors relating to patient characteristics, existing models of service delivery, acceptability and mechanisms of action of group clinics on clinical outcomes)?

## Methods and analysis

### Theoretical and conceptual framework

Development and evaluation of new models of healthcare are frequently hindered by lack of robust, appropriate and explicit theoretical frameworks.^32^ The TOGETHER project will draw on a broad set of substantive social science and educational theories, including:

- ecological theories on supported self-management^33–35^
- work on patient expertise and experiential knowledge in practice^36 37^
- critical education theory and experiential learning^38 39^
- the concept of interdependency in figurational sociology^40 41^

The study will also draw on key theoretical and practical approaches to participatory research that allow researchers, practitioners and service users to learn together for the benefit of service re-design:^42 43^

#### (A) Participatory co-design

The group clinics intervention will be iteratively co-designed using a participatory approach to ensure cultural, developmental and practical relevance; enhance recruitment and retention; and attempt to instigate system change and support sustainability.^44^

#### (B) Participatory research and evaluation

The ‘researcher in residence’ model will be adopted, as a practical manifestation of a participatory approach to research and evaluation. The model has three defining features: the researcher is an integral member of the front-line implementation team, their theoretical and practical contribution is explicit, along with their role to negotiate different bodies of expertise.^45^ The embedded researcher will bridge the qualitative and quantitative evaluations, helping to include practitioner and patient views into the design and feeding back early findings to stakeholders. The model has been applied successfully in a number of different settings.^45 46^ A balance will be sought between how the ‘researcher in residence’ will be contributing to co-design and implementation in practice, and how this involvement will be translated in theoretical terms to increase transferable learning.

### Research plan

The study will be conducted in three phases. A realist review will synthesise findings from existing literature to understand how group clinics may work for young adults with diabetes and other complex needs (phase 1). This understanding will be used to support a participatory co-design process with service users (phase 2). Following implementation of group-based care, the model will be evaluated using qualitative methods and quantitative methods (phase 3).

**Figure 1:**
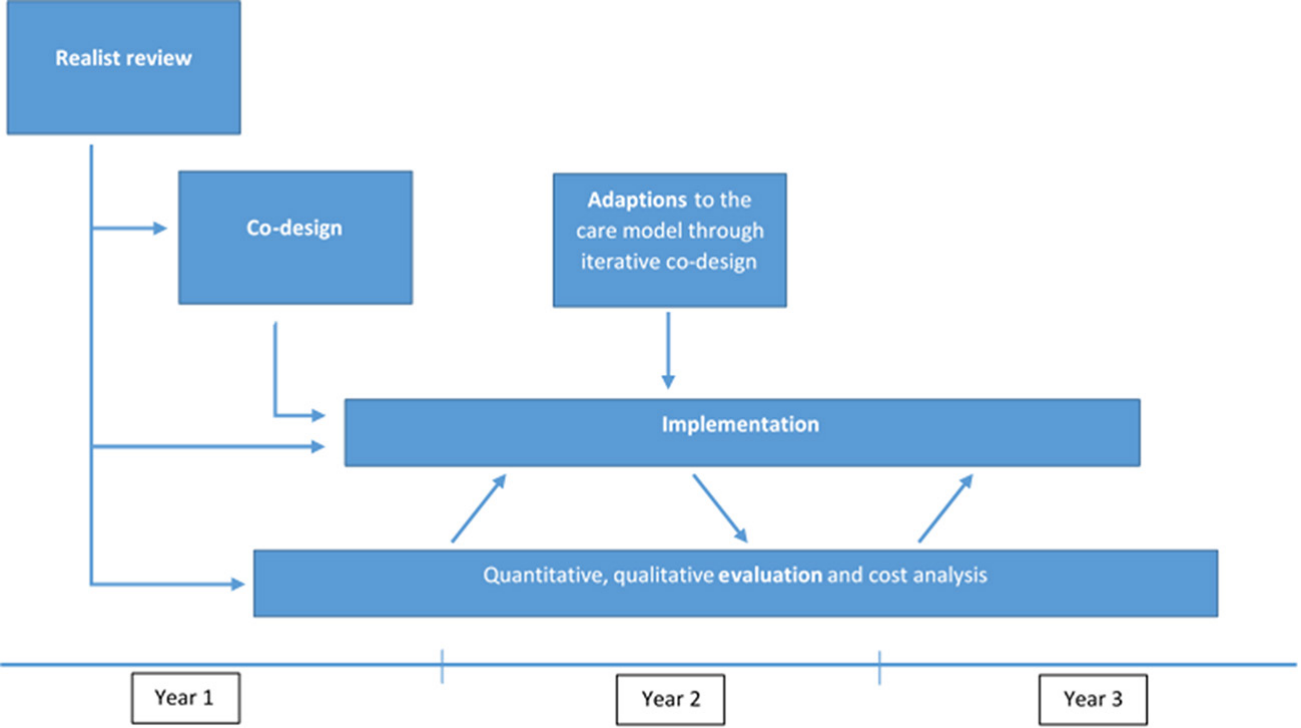
Overview of study design.

The timeline of the project and the processes followed are illustrated in figure 1. The project is funded by the National Institute for Health Research Health Services and Delivery Research programme (ref. no. 15/25/20) to run for 3 years, from December 2016 to November 2019.

### Phase 1: realist review

Following the Realist And Meta-narrative Evidence Syntheses: Evolving Standards (RAMESES), we will undertake a realist review to understand ‘what works, for whom, under what circumstances’ in group clinics for young adults with diabetes.^47–49^ The review will enable us to synthesise data from existing qualitative, quantitative and mixed-methods studies relevant to this topic area. Findings will support co-design, allow context-sensitive tailoring of the intervention and guide implementation and evaluation.^50^

The output of the review will be a realist programme theory expressed in the form of Context–Mechanism–Outcome Configurations (CMOCs) (see Glossary in supplementary files for definitions). We anticipate being able to examine specific mechanisms by which group clinics are deemed to ‘work’, including the potential for the group to generate a sense of greater security for individuals and to create norms, to harness social conformity and peer influence positively to change behaviour and to facilitate experiential learning and social support for self-care and engagement. To do this, we will build on recently completed realist reviews in the area^51 52^ and will extend their findings for a young adult population with complex needs in socioeconomically deprived backgrounds.

The synthesis will follow five iterative stages: (A) locating existing theories, (B) searching for evidence, (C) selecting articles, (D) extracting and organising data and (E) synthesising the evidence and drawing conclusions.^53^

#### (A) Locating existing theories

An exploratory search of the literature along with articles provided by content experts will allow the development of an initial programme theory, that is, a set of provisional assumptions about how group clinics work for young adults with diabetes. This programme theory will continue to be refined throughout the review.

#### (B) Searching for evidence

The formal search strategy will be structured with the help of an information specialist and will be tested in MEDLINE to reach appropriate sensitivity and specificity. Free-text and indexing search terms will include those used by previous systematic reviews.^51 52^ We will search the following databases: Embase, MEDLINE, MEDLINE-in-process, PsycINFO, Web of Science, CENTRAL, Cochrane Database of Systematic Reviews, HTA database and ASSIA. Relevant studies will also be identified by hand searching, forward and backward citation tracking of key sources, contribution of key experts and grey literature where relevant.^54^ Results will be exported to Endnote for de-deduplication using automated and manual checking.

To respond to emerging findings (eg, on the importance of peer influence), we will carry out a second search as the review progresses to ensure we have covered all areas critical to continued engagement of young people in group clinics. This will allow us to progressively focus the review and enhance the explanatory depth of the analysis. Refinement of the programme theory will be discussed in research, co-design and project advisory meetings. This will allow us to link the review to practical questions that are critical in real world settings and provide added value to service delivery.

#### (C) Screening and selecting articles

Studies will be included if they focus on the introduction and delivery of group-based medical visits, group education and similar care delivery models for young people with diabetes. All study designs will be included across healthcare and community settings. Studies will be excluded if they focus solely on populations with considerable differences to young adults, for example, older people or very young children, if they discuss self-management education without a component of group interaction or if they are written in languages other than English. Inclusion and exclusion criteria will continue to be refined as needed throughout the review, according to best practice for realist reviews.^47^ Articles remaining after full-text screening will be classified according to their potential to contribute to programme theory. At the point of inclusion based on relevance, the trustworthiness and rigour of each study will be assessed as appropriate for each study design.^47^ A 10% random subsample of screening decisions will be reviewed by a second reviewer for consistency.

#### (D) Extracting and organising data

The main reviewer will read all articles classified as highly relevant, will extract descriptive study characteristics and will carry out manual coding for immersion in the data. Full texts will be uploaded on to NVivo V.11 (qualitative data management software) to continue coding in broad conceptual categories and to subsequently apply a realist logic of analysis. This means we will work iteratively to identify sections of text related to Contexts (C), Mechanisms (M), Outcomes (O) and the configurations between them (CMOCs), until theoretical saturation has been reached. In doing this, we will seek to interpret and explain how young adults with diabetes reason about and respond (by way of ‘hidden’ mechanisms) to ‘resources’ becoming available through group clinics and to identify the specific contexts or circumstances where these mechanisms are more likely to be ‘triggered’. Again, a 10% random subsample of coded articles will be reviewed by a second reviewer for consistency and disagreements will be solved by discussion.

#### (E) Synthesising the evidence and drawing conclusions

Coded data on CMOCs will be exported into Word documents to develop the final programme theory and the narrative of the synthesis. By moving between data and programme theory, we will be able to refine our explanations of why certain patterns seem to be occurring in certain contexts and under specific circumstances, related to group-based care. This will involve drawing on substantive theory to enhance the plausibility and coherence of the arguments. The final programme theory will consist of evidence-informed propositions, drawing on the literature, substantive theory, professional and patient expertise. These propositions will form the basis for co-design and implementation of group-based care in phase 2.

### Phase 2: co-design and implementation

#### (A) Co-design

Health services often have limited success in changing health-related behaviours unless they take into account the perspectives and priorities of their patients and the staff providing that service.^55^ This is particularly true of patient groups (eg, those defined by age, ethnicity or deprivation) who are poorly served and/or reached by standard care models. The experience-based co-design process^56^ will bring together patients and staff into every stage of the project, supported by experienced co-design facilitators, allowing direct collaboration with the team who will be implementing the care models and regular opportunities for review and iteration. In this way, the co-design process will facilitate the development, review and refinement of user-centred services and care pathways. Co-design will be preceded by a brief national scoping exercise via professional groups to investigate current use and perceptions of group clinics in diabetes care, along with a formative evaluation to map existing local services for young adults with diabetes at two NHS Trusts.

We will recruit 15–20 young adults living with diabetes, members of their support network (eg, parents) and other stakeholders (eg, youth workers and healthcare professionals) to help us develop the group clinic model by drawing on their personal experience in co-design workshops. Participants will be identified via the diabetes service at Barts Health NHS Trust (Newham University Hospital), GP practices and lay support groups. Written information will be given explaining the study and what participation would involve. Co-design workshops will take place in community-based facilities in the London Borough of Newham, a deprived, ethnically diverse population with a high prevalence of long-term conditions and reduced life expectancy compared with UK averages.^57^

#### (B) Implementation

Once the co-designed model of care is established, all patients aged 16–25 years attending the Barts Health NHS Trust (Newham University Hospital) diabetes service will be offered the opportunity to attend group clinics. The 16–25 year age range incorporates adolescents and young adults with both type 1 and type 2 diabetes who are under the care of the multidisciplinary adult diabetes service during and after transition from paediatric services. The adult diabetes service involves close integrated working with local primary care and will facilitate referral into the group clinic model from GP care where relevant. We aim to recruit 80 young adults to this part of the research, using additional sites if required, giving a sample size that will enable sufficient development and evaluation of the new group clinic model, will allow feasibility testing and will guide a future, scaled-up cluster-randomised trial.

Each group clinic is expected to comprise around 8–10 individuals (young adults living with diabetes) and will be run by the Newham diabetes team, with support from related health and social care organisations. The content of these group clinics will be determined by the co-design process and the needs of the group but is likely to include a range of support for diabetes self-management (as already provided in current one-to-one clinics) and extend to a wider set of issues, including other health and social care needs and parental input where required. The content and running of the group clinics is anticipated to evolve during the 2 years in which they will run, due to refinements and revisions guided by ongoing co-design and participant feedback. The anticipated design of the group clinic model will follow two phases: (1) addition: where the group clinics are offered in addition to routine clinical care, and (2) substitution: to be rolled out after the addition model of group clinics has been implemented and considered to be running smoothly and safely by their participants, the clinical and research team and the project advisory group.

### Phase 3: evaluation

#### (A) Qualitative methods

Qualitative methods will allow the research team to develop a better understanding of the contexts and mechanisms by which group clinics do, or do not, work. They will provide rich data on the experiences of patients in group-based care and their attitudes to managing their diabetes and interactions with health services in the wider context of individual, family, employment and other social factors. We will adopt a process-oriented and formative approach to the evaluation, negotiating the analysis and interpretation of the emerging evidence as implementation unfolds in order to maximise the impact of the intervention. Comparison of group clinics with traditional services will allow the relative merits of the new service to be better understood, given the established evidence that improvement interventions are more successful when practitioners can see a relative advantage over current practice.^58^

### Data collection

Data will be generated through a number of methods:

- Individual and group interviews with service users, group facilitators and practitioners: semistructured interviews will be conducted with a sample of service users including those who drop out of the groups and those who receive standard care. Sample size will be determined by data saturation and is expected to comprise between 20 and 30 participants. Purposive sampling will ensure variation in salient characteristics including type of diabetes, type of clinic attended, ethnicity and language. Sampling will also be driven by the findings of the realist review, as to the characteristics and circumstances that play a role in the success or failure of the group clinic model. Group interviews will be conducted with all group clinic facilitators and practitioners delivering standard care. With participant consent, interviews will be audio-recorded and transcribed. Bilingual health advocates will be used where necessary.
- Observations of group clinics and standard care: a sample of group clinics will be observed using a flexible pro forma to capture clinic characteristics such as session content, context, group dynamics and facilitation style. A sample of standard care consultations will be observed as a point of comparison. Detailed field notes will be kept during observations and audio recordings may be taken with participant consent. Group clinics and standard care appointments will be sampled to capture maximum variation (eg, morning/evening clinics in different areas).
- Documents: all documentation produced during co-design sessions, steering group meetings and group clinics will be collected for analysis. Service users and group facilitators will be asked to document their experiences inside and outside of clinics using photos, videos, other visual representations (eg, diagrams and drawings) and/or reflective journals.

#### Data analysis

All data will be analysed thematically using an iterative process of inductive and deductive coding. An initial list of codes will be generated a priori based on the realist review, the co-design stage and our research questions. Codes will be added to this initial list inductively as necessary. Data will be managed using NVivo V.11.

In combination with thematic analysis, the embedded researcher will apply a realist logic of analysis to the data to refine the realist programme theory deriving from the review in phase 1. The programme theory will provide a platform for combining qualitative and quantitative data, primarily in relation to how quantitative outcomes can be linked to qualitatively described processes of change. This will allow us to address the question of how and why group clinics may work differently compared with standard care.

Emerging findings will be discussed with the wider research and co-design group so that the analysis will be co-created with practitioners and service users. Rigour will be enhanced by checking for negative cases that challenge emerging interpretations of the data.

#### (B) Quantitative methods

The detailed qualitative evaluation of the new care model will be complemented by a quantitative evaluation, which will investigate the potential impact of group clinics on clinical outcomes, processes and costs. Definitive evaluation of the impact of group clinics is not possible within this study as the intervention is not yet sufficiently developed, nor will it be delivered at a scale powered to investigate differences in clinical outcomes. The quantitative analysis will therefore (1) provide an early indication of the potential effects of group clinics on engagement and acceptability via attendance rates, measures of patient enablement and through use of the Problem Areas in Diabetes (PAID) scale,^59–61^ (2) investigate change in biological markers of diabetes control (eg, HbA_1c_) to guide sample size calculations for a future trial, (3) test feasibility of collecting service-level, resource (eg, staff contact and non-contact time), activity and process data for future unit-level comparisons in a cluster-randomised trial and (4) estimate costs associated with groups clinics compared with standard care.

The cost analysis will collate data on resource use (including routine and unscheduled care use) and will apply national unit costs to estimate staffing, capital and running costs related to running the standard versus group clinics at service level. Further analysis will look at the potential impact on use of other services and associated costs, and thus the extent to which the group clinic model substitutes for, rather than adds to, standard care. This in turn will provide an early indication of any potential for efficiency savings for the NHS. To best inform a future trial, all resource use and cost data will be presented in both aggregated and disaggregated forms and for different scenarios, for example, group clinic costs according to variations in attendance rates.

### Data analysis

There will be different types of quantitative analysis of clinical outcomes and patient enablement:

- Intention to treat: simple descriptive statistics will be used to compare clinical outcomes among young adults with diabetes who were invited to attend the group clinics with controls from the same clinic over the previous year.
- Comparison of baseline characteristics, clinical outcomes and measures of patient enablement among patients who receive the intervention (n=80) with those at additional external young adult diabetes clinics (n=60) where the group clinics will not be implemented. The first stage of this process will complement qualitative findings on which patients are most attracted by the idea of group clinics. We will compare baseline sociodemographic and clinical characteristics of those who accept versus those who decline the opportunity to participate in group clinics. We will then use longitudinal data to compare the trajectories of clinical and enablement measures over the following year for individuals in the accept versus decline groups (difference in difference analysis).
- Unit-level data of clinical outcome (and indirect clinical outcome) data collected from additional external young adult diabetes clinics and National Diabetes Audit data (published annually by NHS Digital). This data analysis will inform a future scaled-up cluster-randomised controlled trial of group clinics by (1) testing the feasibility of unit-level data collection, (2) identifying differences in the case mix of patients attending young adult diabetes clinics, and (3) characterising the clinical and process outcomes of young adults with diabetes under the care of different units.

### Ethics, safety and dissemination

#### Ethics and safety

The study has been approved by the Office for Research Ethics Committees Northern Ireland (reference 17/NI/0019). Standard rules apply for data security, confidentiality and information governance. Informed consent will be sought for ethnographic observations during group clinics, interviews and for accessing routinely collected NHS data on participants. Confidentiality and privacy between group clinic participants will be a priority, and all participants in the group clinics will be asked to sign a code of conduct to ensure that personal information is not shared outside of the group.

### Dissemination plan and project outputs

Dissemination will be an ongoing process throughout the project, including activity at the outset to raise awareness of the project, at the mid-point to sustain interest and to feed back to participants and at the end and beyond to share learning. Our dissemination plan will build on our participatory approach to identify relevant audiences and maximise impact. We expect to target the following groups:

- project participants, user groups and local staff
- wider stakeholder community (including clinical networks)
- policy makers, strategic decision makers and funders
- academic community

The products needed to target these audiences will vary. We will write regular reports summarising our research activity and outputs, and these will be available via publicly accessible portals. We will prepare user-friendly versions of the main findings, briefing statements and policy summaries. Academic outputs will include journal articles in peer-reviewed, open access journals and conference presentations.

## Discussion

The challenge of improving outcomes for young adults with diabetes is particularly evident in ethnically diverse areas with high levels of socioeconomic deprivation and with an increasing burden of type 1 and type 2 diabetes. Traditional models of diabetes care, based on one-to-one clinic appointments with health professionals, do not meet the needs of these populations consistently, who may see their medical care as only one issue in a complex pattern of health and social care priorities. Although the ability to self-manage long-term conditions is increasingly considered to be the optimal means to achieve good health outcomes, this can lead to power struggles between patient and provider.^62^ There is a need to co-design and evaluate new care models that address diabetes care in the context of these wider needs and increasing demands, to offer alternative support in attaining self-management goals and engagement and to improve the experience and clinical outcomes of people living with diabetes.

As previous work has shown, group-based care requires ‘reallocation of tasks, roles, and resources and a change in providers' attitudes from the traditional prescriptive approach to a more empathic role of facilitator’.^63^ This research will explore the different mechanisms that may or may not allow group clinic participants to engage with care that better suits their needs and improves their health outcomes. We will draw on previous literature to consider a range of potentially relevant factors: prior engagement with health services; appointment duration, frequency and flexibility; congruent social, peer and cultural contexts; and closer integration with other health and social care providers.

This study spans the innovation stage (co-design of a new service model) and early testing stage (evaluation of the acceptability, feasibility, costs and mechanisms of action of group clinic models).^64^ In contrast to studies that start with a clear idea of the intervention and evaluate the impact of introducing this intervention, we do not yet know the optimal role of, or the best way to implement, group clinics, especially among these patient groups in this geographical context. The care model for young adults with diabetes we propose to develop will evolve during the course of our study to ensure it is context sensitive and fit for purpose. For these reasons, our evaluation framework will be largely developmental combining qualitative and quantitative methods to best address our research questions. Project outcomes are expected to inform the potential design of a future cluster-randomised controlled trial to evaluate the impact of group clinics on clinical outcomes and cost-effectiveness.

10.1136/bmjopen-2017-017363.supp1 Supplementary material 1

## Supplementary Material

Reviewer comments
