# Supplementary material for: Group clinics for young adults with diabetes in an ethnically diverse, socioeconomically deprived setting (TOGETHER study): protocol for a realist review, co-design and mixed methods, participatory evaluation of a new care model
Source: BMJ Open. 2017 Jun 21;7(6):e017363. doi: 10.1136/bmjopen-2017-017363 (PMC5726054; doi:10.1136/bmjopen-2017-017363)
Supplement: Supplementary material 1 [file bmjopen-2017-017363supp001.pdf]

Glossary

Contexts: settings, structures, environments, conditions or circumstances that trigger behavioural and emotional responses (i.e. mechanisms) for those affected.

Mechanisms: the way in which individuals respond to and reason about the resources, opportunities or challenges offered by a particular programme, intervention or process. Mechanisms are triggered in specific contexts and lead to changes in behaviour.

Outcomes: impacts or behaviours resulting from the interaction between mechanisms and contexts.

Context-Mechanism-Outcome Configurations (CMOCs): relationships between the building blocks of realist analysis, i.e. how mechanisms are triggered under specific contexts to result in particular outcomes.

Programme theory: a set of theoretical explanations or assumptions about how a particular programme, process or intervention is expected to work.
