# Supplementary material for: Group clinics for young adults with diabetes in an ethnically diverse, socioeconomically deprived setting (TOGETHER study): protocol for a realist review, co-design and mixed methods, participatory evaluation of a new care model
Source: BMJ Open. 2017 Jun 21;7(6):e017363. doi: 10.1136/bmjopen-2017-017363 (PMC5726054; doi:10.1136/bmjopen-2017-017363)
Supplement: Reviewer comments [file bmjopen-2017-017363.reviewer_comments.pdf]

PEER REVIEW HISTORY

BMJ Open publishes all reviews undertaken for accepted manuscripts. Reviewers are asked to complete a checklist review form (**http://bmjopen.bmj.com/site/about/resources/checklist.pdf**) and are provided with free text boxes to elaborate on their assessment. These free text comments are reproduced below.

ARTICLE DETAILS

**TITLE (PROVISIONAL)**	Group clinics for young adults with diabetes in an ethnically diverse, socioeconomically deprived setting (TOGETHER study): protocol for a realist review, co-design and mixed methods, participatory evaluation of a new care model.
**AUTHORS**	Papoutsi, Chrysanthi; Hargreaves, Dougal; Colligan, Grainne; Hagell, Ann; Patel, Anita; Campbell-Richards, Desiree; Viner, Russell; Vijayaraghavan, Shanti; Marshall, Martin; Greenhalgh, Trisha; Finer, Sarah

This article was not externally reviewed at BMJ Open. Protocols that have been independently assessed prior to submission to BMJ Open are usually fast-tracked to publication on the grounds that further substantial changes will not be possible. This independent assessment will usually be external, independent review for both a substantial grant award from a non-commercial or government funder and ethics approval.
